# Mr. Adams on the Cure of Ophthalmia

**Published:** 1813-07

**Authors:** William Adams

**Affiliations:** Oculist Extraordinary to his Royal Highness the Prince Regent, and Oculist in Ordinary to their Royal Highnesses the Dukes of Kent and Sussex. Albemarle-street


### *To the Editors of the Medical and Physical Journal.*

GENTLEMEN,

I FEAR I was not sufficiently explicit in my communication in the 170th Number, page 302, of your valuable Journal, relative to the effects of the Antim. Tartar. in immediately arresting the progress of the purulent inflammation of the conjunctiva, commonly called the Egyptian Ophthalmia, as it appears by a paper from Mr. Fielding, of Hull, in your last Number, that my principle, as well as mode of administering that medicine, have been misunderstood by him. I stated that "constant sickness and vomiting" was kept up for eight or ten hours by repeated doses of Antim. Tartar.:—it would have been more correct if I had said that *violent sickness* and *vomiting* were *continued* during that period.

The diminishing the action of the heart and arteries by *inducing nausea*, is a practice which most professional men have pursued in lessening acute inflammation in various parts; it is not therefore surprising that it should have been tried in a disease where this character is so strongly marked as in the Egyptian Ophthalmia; but I know of *no one* who has recommended *violent vomiting* to be excited and continued in the manner I have described, although it is probable that vomiting may *accidentally* have been produced by small doses of the medicine, when given to *produce nausea.**

The mode of exhibiting my remedy, and the principle on which it is supposed to act, differ so much from those described by Mr. Fielding, that they cannot with propriety be considered the same, or even as analogous. To *produce nausea*, a *quarter*, or at most the *third*, of a grain of the Antim. Tartar. is exhibited for a dose once in *three* or *four hours* to an adult; whereas in my practice I should direct *two grains* to be given at the first, and half that quantity to be repeated every *half hour* until *full vomiting* is produced, which is to be kept up for the stated time, by repeating the dose at longer intervals.

The effect of *nausea* is to lessen arterial action, consequently, during its existence, inflammation in any organ or viscus must be diminished; but I believe its further progress has been very rarely, if ever, *immediately arrested* by so gentle an operation of the medicine. The intentions I had in view in adopting the practice in question were, first, by

* This, however, would be no argument against the originality of my practice, as, had its efficacy been understood by any of those practitioners, they would not, I conceive, have failed systematically to adopt it, as I have done.

the the violent excitement of vomiting to produce a new action in the inflamed vessels, whereby the morbid action constituting the disease would probably be removed; secondly, by keeping up continued sickness and vomiting for so many hours, considerably to exhaust the animal and vital powers, whereby the circulation would become so languid as almost to amount to syncope, during the continuance of which it is impossible that inflammatory action can proceed. By having recourse to the remedy as early as possible before the disease could establish itself, it occurred to me, that not only would the morbid action be removed by inducing a different action, but by the long-continued sickness, and consequent exhaustion, all disposition to a recurrence of inflammatory action would be removed.* The event has most fully answered these expectations, as in no one instance in which I have known the remedy employed agreeably to the rules just laid down, has it failed of success.

After this explanation of the principle and effects of my practice, no candid person, I conceive, will blend it with that recommended by Mr. Fielding, whose liberality I feel assured would not have allowed him to claim priority in its adoption, had leisure permitted me to have been as explicit in my former paper on this subject as I have been at present.

The *nauseating practice* has indeed also been tried by different surgeons in the army, during the period the Egyptian Ophthalmia raged so extensively among the soldiery; and in a report drawn up by three professional gentlemen, appointed by his Royal Highness the Commander in Chief, to inquire into the probable efficacy of my practice, if introduced into the medical practice of the army, they also intimate the two modes of treatment to be similar, although I explained to them, at the conference I had with them on this subject, the important difference both in their intention and effect.

I have the honor to be, Gentlemen,

Your very obedient humble Servant,

WILLIAM ADAMS,

Oculist Extraordinary to his Royal Highness the Prince Regent, and Oculist in Ordinary to their Royal Highnesses the Dukes of Kent and Sussex.

*Albemarle-street*, *June* 13, 1813.

---

* An additional reason for exhibiting the Antim. Tart. as immediately after the accession of Ophthalmia as possible, is, that ulceration of the cornea frequently ensues within twelve hours, (which, by a typographical error in my former paper on this subject was stated twelve *days*,) when vomiting would be highly injurious.

